# Novel heterozygous deletion in the *HNF1β* gene - adolescent with antibody negative diabetes, longstanding hyperglycaemia without ketosis, cataracts, small echogenic kidneys with a cortical cyst, pancreatic atrophy, exocrine pancreatic insufficiency and a uterine anomaly

**DOI:** 10.1186/1687-9856-2015-S1-P1

**Published:** 2015-04-28

**Authors:** Louise S Conwell, Ivan McGown

**Affiliations:** 1 Department of Endocrinology and Diabetes, Royal Children’s Hospital, Brisbane, QLD, Australia; 2 Queensland Children’s Medical Research Institute and School of Medicine, University of Queensland, Brisbane, QLD, Australia; 3 Molecular Genetics Department, Mater Health Services, Brisbane, QLD, Australia

## 

A 13 year old pubertal Caucasian female presented with longstanding polyuria/polydipsia and weight loss of 12kg over 4 months (BMI decreased from 23 to 17.5kg/m^2^ despite a 5cm height gain). There was no personal / family autoimmunity history. A paternal grandparent was thought to have type 2 diabetes. She was haemodynamically stable and mildly dehydrated without clinical signs of insulin resistance. She had bilateral stellate posterior subcapsular cataracts. Plasma glucose was 57mmol/L with normal blood ketones and venous pH. HbA1c was 18.2% (175mmol/mol). Mildly elevated urea and creatinine corrected after rehydration. Liver function tests, serum magnesium and urate were normal. Islet autoantibodies were negative.

Renal ultrasound (US) identified bilaterally small kidneys (<5^th^ centile), with echogenic parenchyma, reduced corticomedullary differentiation, normal cortical thickness and a 5mm left cortical cyst. Urinary albumin/creatinine and calcium/creatinine were normal.

Further imaging (US and MRI) demonstrated severe pancreatic atrophy and a müllerian duct anomaly (arcuate / subseptate uterus).

Faecal elastase 1 in a soft stool sample was low (range of severe exocrine pancreatic insufficiency). However the patient has gained weight with insulin treatment and had normal measures of fat-soluble vitamins.

## Aim

Conduct genetic testing for the autosomal dominant Renal Cysts and Diabetes Syndrome (RCAD), also known as MODY5, caused by heterozygous mutations of the hepatocyte nuclear factor–1 beta (HNF1B) gene (chromosome 17q12).

## Methods

Direct testing for HNF1B gene sequence variations was performed by Multiplex Ligation-dependent Probe Amplification (MLPA) and bidirectional DNA sequencing.

## Results

A heterozygous c390_395del (p.Gln130_Gln131del) was detected. This novel deletion results in the in-frame loss of two glutamine residues in exon 2 of the HNF1B protein. These residues are highly conserved and located in the HNF1 N-terminal domain that contains a dimerization sequence and an acidic region that may be involved in transcription activation. Parental testing has determined that this variant is likely de novo.

## Conclusion

This de novo variant has not been previously described, hence its pathogenicity is unknown. However the phenotype is consistent with RCAD (MODY5).

Written informed consent was obtained from the patient for publication of this Case report. A copy of the written consent is available for review by the Editor-in-Chief of this journal.

